# Epidemiology of perioperative RV dysfunction: risk factors, incidence, and clinical implications

**DOI:** 10.1186/s13741-024-00388-6

**Published:** 2024-04-25

**Authors:** Ben Shelley, Rhiannon McAreavey, Philip McCall

**Affiliations:** 1 Department of Cardiothoracic Anaesthesia and Intensive Care, Golden Jubilee National Hospital, Clydebank, UK; 2 Perioperative Medicine and Critical Care Research Group, University of Glasgow, Glasgow, UK

**Keywords:** Right ventricle, Perioperative medicine, Postoperative complications, Cardiovascular complications

## Abstract

In this edition of the journal, the Perioperative Quality Initiative (POQI) present three manuscripts describing the physiology, assessment, and management of right ventricular dysfunction (RVD) as pertains to the perioperative setting. This narrative review seeks to provide context for these manuscripts, discussing the epidemiology of perioperative RVD focussing on definition, risk factors, and clinical implications. Throughout the perioperative period, there are many potential risk factors/insults predisposing to perioperative RVD including pre-existing RVD, fluid overload, myocardial ischaemia, pulmonary embolism, lung injury, mechanical ventilation, hypoxia and hypercarbia, lung resection, medullary reaming and cement implantation, cardiac surgery, cardiopulmonary bypass, heart and lung transplantation, and left ventricular assist device implantation. There has however been little systematic attempt to quantify the incidence of perioperative RVD. What limited data exists has assessed perioperative RVD using echocardiography, cardiovascular magnetic resonance, and pulmonary artery catheterisation but is beset by challenges resulting from the inconsistencies in RVD definitions. Alongside differences in patient and surgical risk profile, this leads to wide variation in the incidence estimate. Data concerning the clinical implications of perioperative RVD is even more scarce, though there is evidence to suggest RVD is associated with atrial arrhythmias and prolonged length of critical care stay following thoracic surgery, increased need for inotropic support in revision orthopaedic surgery, and increased critical care requirement and mortality following cardiac surgery. Acute manifestations of RVD result from low cardiac output or systemic venous congestion, which are non-specific to the diagnosis of RVD. As such, RVD is easily overlooked, and the relative contribution of RV dysfunction to postoperative morbidity is likely to be underestimated.

We applaud the POQI group for highlighting this important condition. There is undoubtedly a need for further study of the RV in the perioperative period in addition to solutions for perioperative risk prediction and management strategies. There is much to understand, study, and trial in this area, but importantly for our patients, we are increasingly recognising the importance of these uncertainties.

**Supplementary Information:**

The online version contains supplementary material available at 10.1186/s13741-024-00388-6.

In this edition of the journal, the Perioperative Quality Initiative (POQI) present three manuscripts describing the physiology (McEvoy et al. 2023), assessment (Ibekwe et al. 2023), and management (Arora et al. 2023) of right ventricular dysfunction as pertains to the perioperative setting. This narrative review seeks to provide some context for these manuscripts by discussing the epidemiology of perioperative RV dysfunction focussing on its definition, risk factors, and clinical implications.

## Definitions

With the recognition of the critical role of right ventricular function in health and many disease states, it is perhaps unsurprising that the potential for peri-operative RV injury as a cause of morbidity is increasingly being appreciated (Houston et al. 2023). It is these and indeed the POQI authors’ belief (as discussed in the following narrative) that perioperative RV dysfunction is underrecognised, and that ‘if we do not look [for it], we will not see.’ It is important however to know what exactly we are looking for.

The terms RV dysfunction (RVD) and RV failure (RVF) are used ubiquitously in the literature examining peri-operative RV function and injury, but their definitions are often inconsistent. RV *failure* may be easier to define in that it is a clinical diagnosis that is not reliant on any specific imaging or biomarker parameter. A 2018 American Thoracic Society research statement provides a useful working definition in describing RVF as ‘a complex clinical syndrome characterized by insufficient delivery of blood from the RV in the setting of elevated systemic venous pressure at rest or exercise (Lahm et al. 2018)’.

Defining RV *dysfunction* however is more difficult; this term is often used to describe structural changes (abnormal imaging and/or biomarkers) but with maintained cardiac output. In essence, this describes a setting of ‘pre-RV failure’ where, as a result of compensatory mechanisms, cardiac output is maintained but, if the pathophysiological process is not terminated, can progress to RV failure. This concept has sound clinical basis; in the chronic setting in pulmonary hypertension, for example, RVD could describe a period where there is compensation through RV hypertrophy and ultimately pathological dilatation (with associated abnormal imaging and biomarkers) to ensure RV-pulmonary arterial (PA) coupling and cardiac output are maintained. Once these compensatory mechanisms are overwhelmed however, decompensation with RVF and a reduction in cardiac output occur.

In an acute setting such as the peri-operative period, it can be unclear when these ‘normal’ homeostatic mechanisms are overwhelmed and the normal responses of increased venous pressure and RV dilatation, necessary to maintain cardiac output in response to peri-operative insults, become pathological. Further, it is uncertain which of the parameters validated against outcome and used to diagnosis RVD in other clinical conditions (e.g. biomarkers, echocardiography, cardiovascular magnetic resonance imaging, and right heart catheterisation) will have utility in a peri-operative practice.

Beyond clinical examination which can provide information on clinical sequalae of pre-existing RV dysfunction, multiple modalities have been used to explore perioperative RV function, including the following: echocardiography (both transesophageal (Urban et al. 1996; Gouvêa et al. 2022; Schuuring et al. 2013; Denault et al. 2016; Reichert et al. 1992; Levy et al. 2021) and transthoracic (Steffen et al. 2018; Wang et al. 2016)), cardiovascular magnetic resonance imaging (McCall et al. 2019), cardiac biomarkers (McCall et al. 2019), and pulmonary artery catheterisation (Urban et al. 1996; Xu et al. 2014; Segerstad et al. 2019; Reed et al. 1993; Reed et al. 1996; Reed et al. 1992; Okada et al. 1994; Bäcklund et al. 1998; Mageed et al. 2005; Bootsma et al. 2017). As discussed in the POQI ‘assessment’ manuscript (Ibekwe et al. 2023), each technique has its strength and weaknesses, but none is used universally.

## Risk factors

**Fig. 1:**
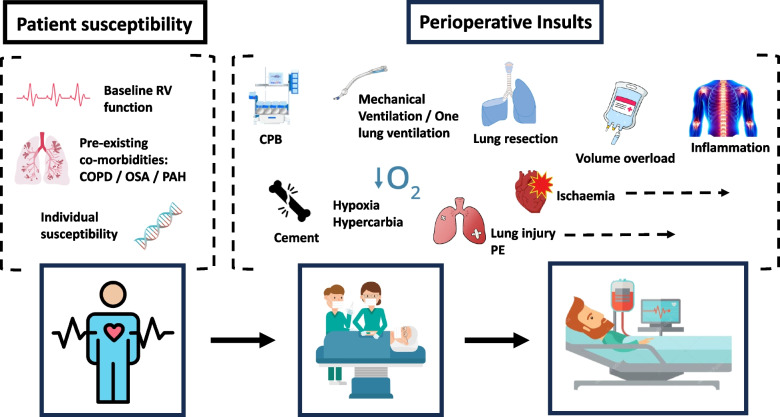
Pre-, intra-, and post-operative risk factors for perioperative right ventricular dysfunction. CPB, cardiopulmonary bypass; COPD, chronic obstructive pulmonary disease; OSA, obstructive sleep apnoea; PAH, pulmonary arterial hypertension; PE, pulmonary embolism; RV, right ventricle

A 2018 scientific statement from the American Heart Association suggests that ‘acute right heart failure may occur during or after noncardiac surgery as a result of the development of acute pulmonary hypertension or intraoperative myocardial ischaemia’ (Konstam et al. 2018). Outside of the cardiac surgical setting however, there has been limited research focussing on RV function in the perioperative period and as such a limited understanding of potential risk factors. It seems likely however that postoperative RVD reflects a complex interplay between pre-existing RVD, patient susceptibility, surgical risk, and a multitude of perioperative insults (Fig. 1).

## Pre-existing RVD

In the general population, RVD is more prevalent in the elderly and in people with hypertension, diabetes mellitus, ischaemic heart disease (IHD), and lung disease (Segerstad et al. 2019), risk factors which are overrepresented in the surgical population. Outside of the cardiac surgical setting, the prevalence of pre-existing RVD in surgical populations has seen limited study; however, what data does exist (Table 1) suggests a prevalence of anywhere between 5.7 and 100% and a profound effect on clinical outcomes. Prevalence figures naturally vary depending on patient population and definition of RVD — in the majority of studies, RVD is defined as ‘normal’ versus ‘abnormal’ on the basis of visual inspection on echocardiography images resulting in a relatively consistent estimate of the prevalence in the region of 5.7–11% (Chou et al. 2021; Chou et al. 2019; Joseph et al. 2021; Meyer et al. 2023). Reflecting an extreme estimate of incidence, Kim et al. however examined RV function in 78 patients with mean age of 80.1 (9.1) years who had sustained a fractured hip and observed that RVD as defined by abnormal RV global longitudinal strain on 2D-speckle tracking was present in all (100%) patients (Kim et al. 2017). Both pre-existing RVD and RV dilatation have been associated with increased incidence of complications and/or mortality in patients undergoing vascular, abdominal, orthopaedic, and renal transplant surgery (Chou et al. 2021; Chou et al. 2019; Joseph et al. 2021; Kim et al. 2017) (Table 1).

**Table 1:** Studies examining the incidence and clinical implications of preoperative right ventricular dysfunction in patients undergoing noncardiac surgery

Study	Surgical population	*N*	Age^A^ Proportion male (%)	Definition of right ventricular dysfunction (all echocardiographic)	Incidence of preoperative RVD	Clinical significance
Kim et al. (2017)	Orthopaedic	78	80.1 (9.1) 24.4%	RVGLS	RVGLS value below the normal range in 100% or patients	RVGLS independently predicted pulmonary complications (*OR* 2.09, 95% *CI* 1.047–4.151, *p* = 0.037)
Chou et al. (2019)	Vascular	108	72 [60-78] 75%	Normal vs. abnormal on visual inspection	10 of 108 (9.3%)	RVD independently associated with post-op major cardiac complications (*OR* = 6.3, 95% *CI* 1.0–38.5, *p* = 0.046) Patients with RVD had a 50% longer LoS (*IRR* 1.5, 95% *CI* 1.2–1.8, *p* < 0.001
Joseph et al. (2021)	Renal transplant	73	51.3 (14.2) 72.5%	Qualitative RV dysfunction and dilatation as adjudicated by the echocardiographer	RVD: 8 of 73 (11%) RV dilatation: 16 of 75 (21%)	RVD: Associated with composite of delayed graft function, graft failure, and all-cause mortality (*p* = 0.026) RV dilation: Associated with a significantly shorter time to all-cause graft failure (*p* = 0.03) and death (*p* = 0.048)
Chou et al. (2021)	Abdominal ‘non-emergent open abdominal surgery’	122	65 [55-74]B 56 [45-68]B 45%	Normal vs. abnormal on visual inspection	7 of 122 (5.7%)	RVD independent risk factor for all‑cause in‑hospital mortality (*OR* 18.9, 95% *CI* 1.8–201.7, *p* = 0.015)
Meyer et al. (﻿^2023^)	Vascular	776	67 [60-75] 68%	Mild, moderate, or severe decrease in RV systolic function (no definition provided)	85 of 776 (11%)	No association between RVD and major adverse cardiovascular events

^A^Presented as *n* (%), mean (standard deviation), or median [inter-quartile range]. ^B^Data presented for two experimental groups separately

*IRR* incidence rate ratio, *LoS* length of stay, *RVD* right ventricular dysfunction, *RVGLS* RV global longitudinal strain

## Susceptible patient groups

### Chronic obstructive pulmonary disease (COPD)

**Fig. 2:**
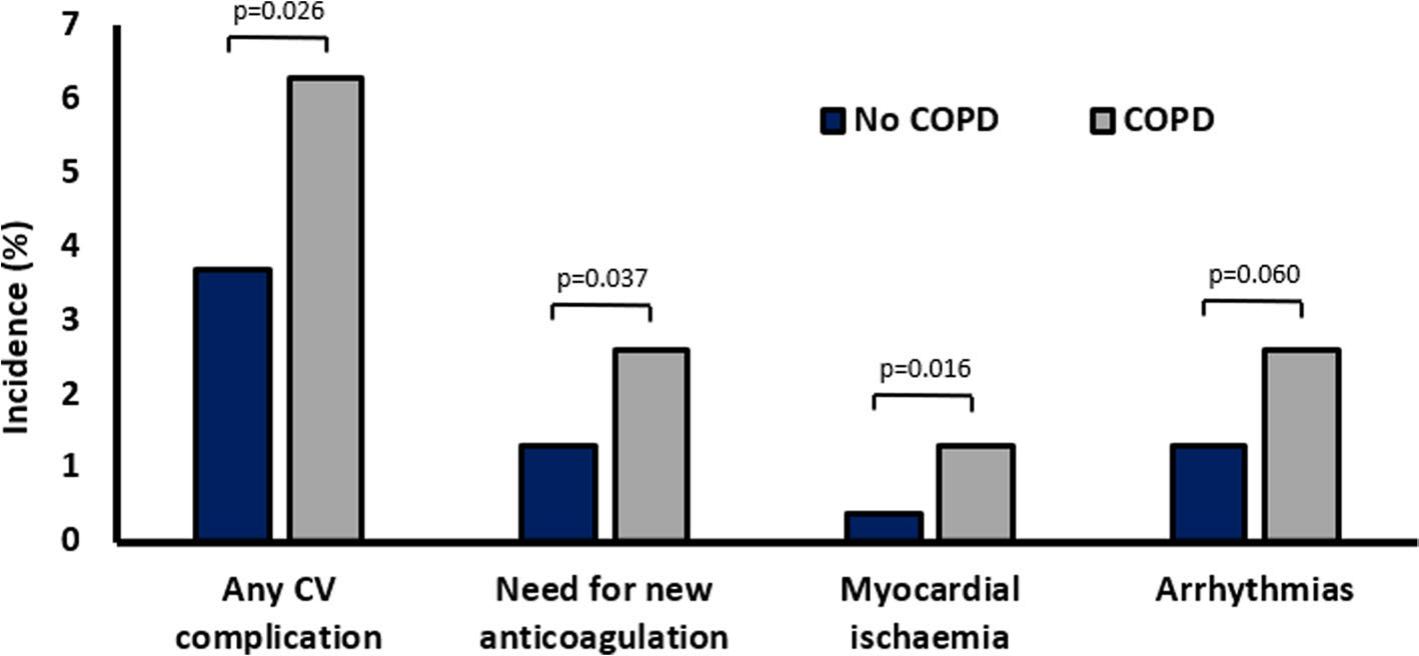
Secondary analysis of the VISION-UK Database by history of COPD demonstrating increased risk of cardiovascular complications in patients with COPD (Ackland et al. 2020)

Patients with moderate to severe COPD per GOLD criteria (i.e. with impaired pulmonary function but not to the extent to preclude surgical candidacy) have significantly reduced RV ejection fraction (RVEF) compared to healthy controls (Gao et al. 2011). Furthermore, in patients with COPD, the stroke volume response to exercise can be limited by inability to reduce pulmonary vascular resistance (PVR) in the face of increased cardiac output (Holverda et al. 2009). Post hoc analyses of 4303 UK patients recruited to the Vascular Events in Noncardiac Surgery Patients (VISION) study reveal that patients with COPD (7% of the overall study cohort) are more likely to incur perioperative myocardial injury (43.5% vs 28.4% in patients without COPD, *p* < 0.001) and more likely to suffer cardiovascular complications (Fig. 2), and that COPD is an independent predictor of postoperative mortality (Devereaux et al. 2017). It is conceivable that some of this increased risk of perioperative cardiovascular complications is mediated by RVD.

### Obstructive sleep apnoea (OSA)

More widespread use of screening tools has revealed the high incidence of (often undiagnosed) OSA in surgical populations (Singh et al. 2013). In OSA, hypoxic pulmonary vasoconstriction occurs during apnoeic episodes leading to remodelling of the pulmonary microcirculation increasing PVR and promoting the development of pulmonary hypertension and subsequent RV dysfunction (Murphy and Shelley 2019). Patients with both unrecognised and diagnosed OSA are well described as being at increased risk of perioperative cardiovascular complications (Chan et al. 2019; Kaw et al. 2012).

### Pulmonary hypertension

RV function is the ultimate driver of survival in patients with pulmonary arterial hypertension (PAH). As RV afterload increases, this is paralleled by an initially adaptive RV remodelling response (characterised by preserved volumes and hypertrophy) followed by a pre-morbid period of mal-adaption (characterised by dilatation, dyssynchrony, and eccentric hypertrophy). A large observational multicentre observational study in the United States examining data from over 17 million patients identified an incidence of PAH of 0.81% in patients referred for major noncardiac surgery (Smilowitz et al. 2019). In this cohort, after adjusting for demographics, clinical covariates, and surgery type, PAH remained independently associated with major adverse cardiovascular events (*aOR* 1.43, 95% *CI* 1.40 to 1.46) (Sanz et al. 2019).

Though the increased risk of postoperative cardiovascular complications being mediated through RV dysfunction in patients with COPD, OSA, or PAH has not been rigorously demonstrated, analogy is commonly drawn between the perioperative period and a prolonged period of exercise such that assessment of exercise capacity is a fundamental facet of perioperative risk stratification. If patients with COPD, OSA, or PAH are limited in their ability to exercise due to impaired RV function, then it is not hard to conceive that their ‘performance’ in the perioperative period might similarly be influenced by RV function.

## Perioperative insults

Though in many scenarios a clear mechanistic link between a potential perioperative ‘insult’ and perioperative RVD has not been demonstrated, there are many clinical scenarios occurring in the perioperative period that have the potential to adversely affect RV function either through excessive preload, direct influence on contractile function, or in many cases increases in afterload.

### Volume overload

There is wide variability in the practice of perioperative fluid administration; whilst fluid administration is deemed necessary in situations where augmentation of perfusion is required and patients are ‘fluid responsive’, there is increasing recognition of the potential harms associated with excessive fluid administration (Navarro et al. 2015). Whilst the RV is classically described as being ‘tolerant’ of pre-load, injudicious fluid administration to the vulnerable RV may result in RV distention, dilatation of the tricuspid annulus, and development or worsening of tricuspid regurgitation. Significant tricuspid regurgitation leads to further volume overload and reduces forward flow. Volume overload of the RV can distort the LV shape and impair LV filling and function reducing systemic cardiac output (Murphy and Shelley 2018).

### Myocardial ischaemia

Whilst symptomatic myocardial infarction is uncommon after noncardiac surgery, large numbers of patients have biochemical evidence of perioperative myocardial injury (PMI) (Devereaux et al. 2017). It is widely hypothesised that PMI results from myocardial oxygen supply/demand imbalance (Devereaux and Szczeklik 2020). In the face of increased afterload (as may occur with mechanical ventilation intraoperatively or in response to perioperative insults (see below)), increased RV intracavity pressure during systole means the distribution of coronary blood flow to the RV during the cardiac cycle is more like that of the LV, occurring only during diastole in contrast to the somewhat luxurious physiological situation of RV perfusion throughout the cardiac cycle (McEvoy et al. 2023; Vlahakes et al. 1981). Such an alteration in coronary flow may predispose to ischaemia in patients with IHD within RV territories. Using advanced oxygen-sensitive cardiovascular magnetic resonance, Guensch et al. recently demonstrated (for the first time) the existence of dynamic changes in myocardial tissue oxygenation and subsequent impaired strain and wall motion abnormalities in the LV (including right coronary artery territories and the septum) during induction of anaesthesia (Guensch et al. 2023). Although due to the reduced muscle bulk of the RV free wall no assessment of RV oxygenation could be made, it is reasonable to hypothesise similar changes in RV tissue oxygenation might occur.

### Pulmonary embolism (PE)

Whilst overt PE is uncommon, subclinical PE occurs frequently in surgical populations. Grobben et al. demonstrated that clinically silent PE was evident in 28% of patients undergoing elective intermediate- to high-risk noncardiac surgery, a finding which was substantially more common in patients with myocardial injury (Grobben et al. 2018).

### Lung injury and inflammation

Pre-existing lung disease and the combined deleterious effects of ventilator induced lung injury, systemic inflammation, and fluid overload mean that subclinical lung injury is detectable in a large proportion of postoperative patients, whilst overt clinical lung injury is not uncommon (O’Gara and Talmor 2018). Lung injury increases RV afterload by a variety of well-described mechanisms including hypoxic vasoconstriction, extrinsic vascular compression as a result of interstitial oedema, vasoconstrictor mediator release, and blood vessel remodelling (Murphy and Shelley 2019).

### Mechanical ventilation

In susceptible patients, such as those with pre-existing RV dysfunction, IHD, or respiratory disease, the increase in afterload associated with institution of (bi-lung) mechanical ventilation may result in acute RV dysfunction. The development of disproportionate haemodynamic instability following intubation in the COPD patient is a classic example of this (Murphy and Shelley 2019).

### One-lung ventilation

A period of one-lung ventilation (OLV) adds an additional haemodynamic challenge; during OLV, there is a near doubling of dependant lung blood flow, a redistribution of flow which has been consistently demonstrated to result in a 25–35% increase in pulmonary artery pressure (PAP) and a 20–50% increase in PVR (Shelley et al. 2023). Haemodynamic adaptation to these conditions of acutely increased afterload relies both on the ability of the pulmonary circulation to accommodate this increased flow, whilst pulmonary vascular flow reserve and the ability of the RV to maintain cardiac output in the face of the ensuing increased afterload (RV contractile reserve). It is likely that in a minority of patients, pulmonary vascular or RV comorbidity results in an inability to adequately adapt (Shelley et al. 2023).

### Hypoxia and hypercarbia

The acute physiological effects of hypoxia and hypercarbia causing pulmonary vasoconstriction and increased PVR are well described (West 2005). Further, both hypoxia and hypercapnia may have a direct negatively inotropic effect on the myocardium (Than et al. 1994). There is however some uncertainty regarding the clinical implications of such changes in the perioperative period, with little structured investigation examining their independent effects on RV afterload. In healthy volunteer models of hypercapnia induced by carbon dioxide rebreathing, PAP and PVR are increased, but these effects are compensated by increased heart rate and stroke volume resulting in a net increase in cardiac output (Kiely et al. 1996). Similarly, in experimental models of hypoxia (often examined in the context of altitude), though mild pulmonary hypertension is demonstrated, this is easily compensated (Naeije and Dedobbeleer 2013). These examples however reflect the compensatory mechanisms seen in normal physiology; it seems plausible (and indeed anecdotal experience suggests) than in the face of exhausted compensatory mechanisms, even modest increases in afterload may be sufficient to trigger decompensation.

### Lung resection

Though intuitive, the hypothesis that postoperative RV dysfunction stems from increased afterload caused by mechanical obstruction to blood flow in a reduced capacity vascular bed has not been well demonstrated. Whilst intraoperatively pulmonary vascular resistance increases on institution of OLV and at pulmonary artery clamping, this acute increase returns to baseline postoperatively (Lewis et al. 1994; Waller et al. 1996), yet RV function remains depressed (McCall et al. 2019). More recent work however has demonstrated profound changes in pulsatile afterload quantified in terms of pulse wave reflection and pulmonary artery compliance following lung resection which are persistent postoperatively and are associated with reduced RVEF (Glass et al. 2023).

### Medullary reaming and cement implantation

Bone cement implantation syndrome (BCIS) refers to a clinical syndrome characterised by hypoxia, hypotension, cardiac arrhythmias, increased PVR and cardiac arrest which occurs following femoral reaming, acetabular or femoral cement implantation, insertion of the prosthesis, or joint reduction during total hip joint replacement (Donaldson et al. 2009). Embolic showers have been detected using echocardiography in the right atrium, RV, and pulmonary artery (Donaldson et al. 2009; Bisignani et al. 2008). Whilst increases in RV afterload (Urban et al. 1996; Segerstad et al. 2019) and on surveillance visualisation of the passage of echogenic embolic material is relatively common place (Bisignani et al. 2008), clinically significant RV dysfunction is less common. Across all types of arthroplasty, the incidence of severe BCIS (characterised as severe hypoxia or hypotension, unexpected loss of consciousness, or cardiac arrest) is estimated to occur in 5.7% of cases (Rassir et al. 2021).

### Cardiac surgery and cardiopulmonary bypass

Cardiac surgery presents a high risk for perioperative RV dysfunction and failure with multiple potential insults occurring to influence preload, contractility, and afterload. This is often coupled with a high prevalence of pre-existing RVD, often related to the indication for surgery, pulmonary hypertension (secondary to left-sided valvular disease), right-sided valvular disease for repair/replacement, right-side coronary artery disease with ischaemia, atrial and ventricular septal defects, pericardial disease, and pericardial effusions/tamponade. Peri-operatively, there is risk of volume overload (excessive transfusion), myocardial dysfunction (direct myocardial injury, hypotension, pre-existing cardiomyopathy, ischaemia (including air embolus to right coronary artery) and suboptimal myocardial protection), and increased RV afterload (from pulmonary atelectasis, ischaemia/reperfusion, protamine reaction, pulmonary embolism, and dynamic RV outflow tract occlusion) (Estrada et al. 2016; Jabagi et al. 2022). Further, the high peri-operative risk of bleeding, along with the cardiac and systemic inflammatory effects of cardiopulmonary bypass (CPB) resulting in myocardial dysfunction and vasodilatation/vasoplegia, can compound these perioperative risks.

### Cardiac transplantation and left ventricular assist device implantation

Cardiac transplantation and left ventricular assist device implantation are further extreme examples of perioperative insults, in addition to those above, which can result in perioperative RV dysfunction (Zochios et al. 2023). Risk factors are classified as donor, recipient, or procedural (Kobashigawa et al. 2014). In cardiac transplant, the heart undergoes a series of insults which begins with the donor, where the autonomic storm following brain death (in donation following brain death (DBD)) leads to RV dysfunction which persists following implant (Trigt et al. 1995; Bittner et al. 1999). There is a growing interest in donation following circulatory death (DCD), and given the requirement for cardiac arrest, it may seem intuitive there is increased risk of cardiac dysfunction in this cohort. In hearts transplanted following DCD, there is evidence of increased incidence of transient post-operative RVD (when compared to a DBD cohort), which resolves by 3 weeks (D'Alessandro et al. 2022). Donor-recipient size matching is critical, with size mismatch (smaller donor hearts implanted in to larger recipients) being associated with an increased risk of RVD, particularly in those recipients with pre-existing pulmonary hypertension (Shah et al. 2020). Organ procurement and preservation technique along with ischaemic time (Ahlgren et al. 2011), and manual handling, can all contribute to increased risk of RVD. In addition, the recipient often has a degree of pulmonary hypertension as a result of end-stage heart failure. When the ‘afterload naïve’ donor heart is implanted, this combination can result in physiological conditions where RVD is likely to occur.

RVD often complicates the course of patients undergoing LVAD implantation and can have a significant impact on outcomes (Kapelios et al. 2022; Kormos et al. 2010). Patients often have a degree of pre-existing RVD, and although benefiting from the reduction in left atrial (and thus pulmonary artery) pressure from ‘offloading the LV’, the restored cardiac output can lead to RV volume overload with subsequent dilatation and ischaemia. In addition, geometric distortion resulting from LVAD restored cardiac output can lead to a shifted interventricular septum compromising the LV contribution to RV contractility (Zochios et al. 2023; Bravo et al. 2022; Argiriou et al. 2014; Lo Coco et al. 2021).

### Lung transplantation

Many of the risk factors previously described are important for patients undergoing lung transplantation. PAH remains a primary indication for transplant, and international registry data demonstrate secondary pulmonary hypertension associated with lung disease is common in patients with advanced cystic fibrosis, idiopathic lung disease, and COPD with impact on oxygen requirements and survival (Leard et al. 2021). These important pre-op factors have important implications in the peri-operative management of these patients, with the insult of general anaesthesia, positive pressure ventilation (particularly with OLV), and PA clamping leading to significant cardiovascular instability (Marczin et al. 2021; Tomasi et al. 2018).

## RVD following noncardiac surgery

### Incidence

A statement from the American Heart Association suggests ‘that the prevalence of right heart failure after noncardiac surgery is difficult to determine’ (Konstam et al. 2018); in reality, there has been little systematic attempt at quantification. There are however a number of isolated reports which suggest that when specifically sought, postoperative RVD can be found not infrequently (Table 2). Once again however, these reports are challenged by the definitions of RV function used and the methods of RV assessment employed. Impaired RV dysfunction has been demonstrated via a variety of differing assessment modalities in patients undergoing thoracic (Steffen et al. 2018; Wang et al. 2016; McCall et al. 2019; Reed et al. 1993; Reed et al. 1996; Reed et al. 1992; Okada et al. 1994; Bäcklund et al. 1998; Mageed et al. 2005; Elrakhawy et al. 2018), orthopaedic (Urban et al. 1996; Segerstad et al. 2019), oesophageal (Xu et al. 2014), and liver transplant surgery (Gouvêa et al. 2022). It is noteworthy however that the majority of this literature has been generated using ‘fast-response’ pulmonary catheters, a technology the validity of which has increasingly been called into question (Leibowitz 2009; Bootsma et al. 2022). Regrettably, such an observation weakens an already limited evidence base.

**Table 2:** Summary of studies describing post-operative changes in right ventricular function and their clinical significance in patients undergoing noncardiac surgery

Study	Surgical population	*N*	Age Proportion male (%)	Method of assessment	Definition of RVD	Postoperative change	Clinical significance/comments
Reed et al. (1992)	Thoracic (lobectomy and pneumonectomy)	15	65 (1.8) 80%	PAC	RVEF RVEDV	Early post-op vs POD2: • RVEF: 0.40 (0.01) to 0.36 (0.03) • RVEDV: 153 (10) to 173 (14)	‘Three patients had periods of sustained atrial arrhythmias on POD 1 or 2 and at the time had significant increases in RVEDV’
Okada et al. (1994)	Thoracic (predominantly lobectomy)	20	62 (49-77) 90%	PAC	RVEF RVEDV	Preop vs POD1 vs 3 weeks: • RVEF: 0.43 (0.07) to 0.36 (0.04) to 0.36 (0.34) (*p* < 0.05) • RVEDV increased on POD2: 112 (20) vs 130 (24) ml/m^2^ (*p* < 0.05)	RVEF remained depressed 3 weeks post-op
Urban et al. (1996)	Orthopaedic (revision THJR)	18	41–88 Not provided	PAC TOE (in some)	Decrease in RVEF ≥ 10% and increase PAP ≥ 10 mmHg	RVD in 4 of 18 (22%) at end of surgery	Transient increase in inotropic support. Mortality in one patient ‘complications related to bone cement implantation syndrome’
Xu et al. (2014)	Oesophagectomy	40	59.0 (7.8) A 60.6 (6.6) A 73%	PAC	RVEF	Approx. 5% reduction overall at end of surgery	Not examined
Wang et al. (2016)	Thoracic (pneumonectomy and lobectomy)	30	53.1 ± 10.7 A 57.0 ± 11.4A 73%	TTE	RVFWLS RVGLS	All pre-op vs 1-week post-op: Pneumonectomy • RVFWLS: − 30.86 (5.88) to − 11.77 (4.14) • RVGLS: − 24.56 (5.32) to − 12.04 (5.33) Lobectomy • RVFWLS: − 29.7 (6.23) to − 18.03 (8.06) • RVGLS: − 25.69 (4.71) to − 17.07 (5.26) *p* < 0.05 for all	Not examined
McCall et al. (2019)	Thoracic (anatomical lobectomy)	27	67 (59-74) 37%	CMR	RVEF	RVEF deteriorated from 50.5% (6.9) pre-op to 44.9 (7.2) on POD2 (*p* = 0.003)	RVEF on POD2 associated with length of postoperative critical care unit stay (*r* = − 0.653, *p* = 0.001) RVEF remains depressed vs. baseline 3-month post-op
Segerstad et al. (2019)	Orthopaedic (THJR)	22	76 (8.1) A 74 (6.2) A 36%	PAC	RVEF	8% reduction in cemented, unchanged in uncemented	Not examined
Gouvêa et al. (2022)	Liver transplantation	19	52 (13)	TOE	TAPSE < 17 mm or FAC < 35%	No change at end of surgery	Right ventricular function was found to be normal throughout the procedure

Data presented as proportion of patients exhibiting predefined reduction in RVD parameter or as change in a continuous parameter as described in the original paper

^A^Data presented for two experimental groups separately

*CMR* cardiovascular magnetic resonance, *FAC* fractional area change, *NS* non-significant, *PAC* pulmonary artery catheter, *PAP* pulmonary artery pressure, *POD* postoperative day, *RVD* right ventricular dysfunction, *RVEDV(I)* RV end-diastolic volume (index), *RVEF* RV ejection fraction, *RVFWLS* RV free wall longitudinal strain, *RVGLS* right ventricular global longitudinal strain, *TAPSE* tricuspid annular plane systolic excursion, *THJR* total hip joint replacement, *TOE* transoesophageal echocardiography, transthoracic echocardiography

Due to the obvious profound manipulations of the pulmonary vasculature, RV function after noncardiac, thoracic surgery involving lung resection has been the subject of a greater quantity of research. In this group, there is a consistent decrement in RVEF postoperatively of between 3 and 10% (Table 2 and Supplementary Table 1) — whilst much of this literature has also been generated using fast-response pulmonary artery catheters, these changes have since been confirmed using gold-standard cardiovascular magnetic resonance (McCall et al. 2019)). Though most commonly examined in the immediate postoperative period, such dysfunction has been demonstrated to persist weeks (Okada et al. 1994) and months (McCall et al. 2019) following surgery. Importantly, whilst the (mean) impairment in RVEF at rest might be considered modest, the limited number of studies that have examined dynamic RV function on exercise reveals a more pronounced effect suggesting a loss of RV contractile reserve postoperatively (Okada et al. 1994; McErlane et al. 2023).

## Clinical implications

Whilst mortality and significant morbidity are easily recognised sequelae of major surgery, it is increasingly recognised that overt complications are the ‘tip of the iceberg’, and that a significant burden of covert postoperative complications exist and have significant long-term impact (Ludbrook 2018). Acute manifestations of RVD mainly result from low cardiac output or systemic venous congestion, leading to kidney injury, gut oedema, liver dysfunction, and cerebral oedema, all of which are non-specific to the diagnosis of RVD (Murphy and Shelley 2019). As such, RVD is easily overlooked, and the relative contribution of RVD to postoperative morbidity is likely therefore to be underestimated. Elegantly reflecting the hypothesis that if sought evidence of postoperative RVD is found more commonly than appreciated, Markin et al. analysed the findings of 364 ‘rescue’ echocardiograms performed in cases of severe perioperative haemodynamic instability. In this mixed surgical cohort (only 20% of whom had cardiac surgery), RVD was identified with equal frequency to LV dysfunction (Markin et al. 2015). Rescue echocardiography was defined as ‘any examinations ordered by a perioperative physician on an urgent/emergent basis for a patient with hemodynamic instability’; in such circumstances, RVD was identified in 9.9% of unstable patients examined intraoperatively and 24.1% of patients examined postoperatively.

Few studies however have specifically examined the clinical impact of acquired postoperative RVD (distinct from pre-existing RVD discussed above). Impaired RVD in the postoperative period has been associated with atrial arrhythmias (Reed et al. 1992) and prolonged length of critical care stay following thoracic surgery (McCall et al. 2019) and increased need for inotropic support in revision orthopaedic surgery (Urban et al. 1996) (Table 2).

## RVD following cardiac surgery

### Incidence

As a result of the high frequency of RV dysfunction/failure in patients presenting for cardiac surgery and the multitude of associated insults that can occur to the RV peri-operatively, the importance of RV function in this cohort of patients is better recognized. Further, the cardiac anaesthetist is afforded the luxury of visualisation of RV function (by transesophageal echocardiography or direct observation of the surgical field) in real time. Following cardiac surgery, RVF may manifest intraoperatively as difficulty weaning from cardiopulmonary bypass and postoperatively with low cardiac output and end-organ dysfunction. In contrast to the noncardiac surgery population, there has been a drive to better understand the incidence and implications of RVD/RVF in this population. Criteria used in this context include clinical parameters (difficulty weaning from CPB), echocardiographic parameters, and pulmonary artery catheter-derived variables.

In patients undergoing cardiac surgery, as a result of variability in both baseline and procedural risk, along with wide variation in diagnostic criteria used, the incidence of RV dysfunction/failure varies widely and ranges from 0.04 to 34.6% (Table 3). Although consistency is lacking, there have been efforts to try and create a standardized perioperative definition of RVF in this patient cohort (Table 4) (Jabagi et al. 2022).

**Table 3:** Summary of selected studies describing post-operative changes in right ventricular function and their clinical significance in patients undergoing cardiac surgery

Study	Surgical population	*N*	Age Proportion male (%)	Method of assessment	Definition of RVD	Incidence of postoperative RVD	Clinical significance/comments
Reichert et al. (1992)	Cardiac surgery — mixed	52	Not stated	Clinical and echo	Hypotension (< 65 mmHg) despite inotropes + / − IABP and *RVFAC* < 35%	Evidence of RVF in 18 (34.6%) • 9 (17.3%) were biventricular failure • 9 (17.3%) isolated RVF	Mortality 81.8% in biventricular failure and 90% in isolated RVF
Maslow et al. (2002)	Cardiac surgery — CABG with severe LVSD (*LVEF* < 25%)	41	61.4, 56.3–66.5 85.4%	Echo	*RVFAC* < 35%	7 (17.1%)	Associated with early (30 days) mortality (71% vs 0) and prolonged duration of mechanical ventilation and both ICU and hospital stay
Moazami et al. (2004)	Cardiac surgery — mixed	9270	58 (15) 13 (43.3%)	Clinical	Need for RVAD	30 (0.3%) need for RVAD	Mortality 66.6%. Excluded medically managed RVF
Schuuring et al. (2013)	Cardiac surgery — congenital heart disease	412	36, 18–74 56%	Clinical and echo	‘Elevated jugular venous pressure’, impaired RV function on echo and a diagnosis of RV failure documented in the medical charts	4.4%	Mortality of 33.3% in RV failure group vs 2.3% in non-RV failure group (*p* < 0.01) Impaired pre-op RV function, SVT and CPB time associated with post-op RV failure
Denault et al. (2016)	Cardiac surgery — high risk with pulmonary hypertension	124	68.3 (9.2)A 70.2 (10.2)A 48.4%	Clinical and echo	Hemodynamic instability, defined as difficult or complex separation from CPB, 20% reduction in RVFAC, and visualisation of impaired or absent RV wall motion	18 (14.5%)	Mortality 22% in RVF group vs 2% in no RVF (*p* < 0.001)
Bootsma et al. (2017; Bootsma et al. 2018)	Cardiac surgery — mixed	1109	74 [67-79]A 70 [63-77]A 66 [58-73]A 64.8%	PAC	RVEF < 20% within first 24 h	216 (19.5%)	RVF associated with 2-year mortality — 16.7% vs 8.2% vs 4.1% in those with RVEF < 20%, 20–30% and > 30% respectively (*p* < 0.001). RVEF associated with ICU LOS, duration of mechanical ventilation, and increased creatinine
Levy et al. (﻿^2021^)	Cardiac surgery — mixed	3826	68.6 (10.9) 74.5%	Clinical and echo	Hemodynamic instability requiring vasoactive support and immediate post-op pulmonary vasodilators with echo evidence of RVF; RV free wall hypokinesia or IVS flattening or RV dilatation (RV/LV ratio > 1)	110 (2.9%)	No difference in mortality (1.8% vs 0.7%). RVF associated with post-op AF and ICU LOS

Presented as *n* (%), mean (standard deviation), median [interquartile range] or median range. ^A^Data presented for separate experimental groups

*AF* atrial fibrillation, *CABG* coronary artery bypass grafting, *CPB* cardiopulmonary bypass, *IABP* intra-aortic balloon pump, *ICU* intensive care unit, *IVS* interventricular septum, *LOS* length of stay, *LVEF* left ventricular ejection fraction, *LVSD* left ventricular systolic dysfunction, *PAC* volumetric pulmonary artery catheter, *RVAD*, right ventricular assist device, *RVF* RV failure, *RVFAC* RV fractional area change, *SVT* supraventricular tachycardia

**Table 4:** Proposed definition of perioperative right ventricular failure in patients undergoing cardiac surgery

A. Intraoperative acute RVF	
i) Difficult separation from CPB, characterized by either the following:	
1	Concurrent use of ≥ 1 vasopressor and ≥ 1 inotrope and/or inhaled pulmonary vasodilator **OR**
2	Requiring > 1 CPB weaning attempt for RVF OR
3	Mechanical support device to facilitate wean (i.e. IABP or RVAD)
**AND**	
ii) Anatomical visualization of impaired or absent RV wall motion by the following:	
a	Direct intraoperative visual inspection* **OR**
b	> 20% reduction in RVFAC measured by 2D echocardiography
**OR**	
**B. Postoperative acute RVF (haemodynamic criteria on arrival to ICU)**	
i	*CVP* > 15 mmHg or *CI* < 1.8 Lmin-1 m-2 **AND**
ii	Absence of elevate LAP and PCWP > 18 mmHg, tamponade, VT, or pneumothorax** **AND**
iii	RVSWI < 4 where *RVSWI* = 0.136 × SVI × (mPAP-RAP), and SVI = strove volume/BSA

Adapted from Jabagi et al. (Jabagi et al. 2022)

^*^Global hypokinesis or akinesis and/or severe RV dilatation/ballooning

^**^Causing haemodynamic compromise or tension pneumothorax

*RVF* right ventricular failure, *CPB* cardiopulmonary bypass, *IABP* intra-aortic balloon pump, *RVAD* right ventricular assist device, *RVFAC* right ventricular fractional area change, *CVP* central venous pressure, *CI* cardiac index, *LAP* left atrial pressure, *PCWP* pulmonary capillary wedge pressure, *VT* ventricular tachycardia, *RVSWI* RV stroke work index, *SVI* stroke volume index, *mPAP* mean pulmonary artery pressure, *RAP* right atrial pressure, *SVI* stroke volume index, *BSA* body surface area

For patients undergoing cardiac transplantation, the International Society for Heart and Lung Transplantation (ISHLT) developed consensus definitions for primary graft dysfunction (PGD) (including PGD-RV) in 2014 (Kobashigawa et al. 2014). Limitations have been highlighted with these criteria in real-world practice, as they are often limited to the most severe form of RVF requiring RV assist device implantation, potentially underestimating the incidence (Alam et al. 2020). Using ISHLT criteria, the incidence of PGD-RV has been reported from 1 to 12.3% (Cosío Carmena et al. 2013; Avtaar Singh et al. 2019; Nicoara et al. 2018), but when using alternative definitions, the incidence has been reported as high as 59% (Kaveevorayan et al. 2023) (Supplementary Table 2).

Following LVAD implantation, one of the most significant drivers of postoperative morbidity and mortality is RV failure. The definition of RV failure following LVAD implantation has developed from the first iteration of the Interagency Registry for Mechanically Assisted Circulatory Support (INTERMACS) definition in 2008. This was updated in 2014 and most recently has been surpassed by the 2020 Mechanical Circulatory Support Academic Research Consortium (MCS-ARC) definition (Kormos et al. 2020). This revision incorporates clinical and haemodynamic findings, is focused on timing from LVAD implantation and acuity of up-escalation of mechanical or nonmechanical support, and is thought to be more sensitive for disease recognition. Post-LVAD implantation RV failure is discussed as occurring at three timepoints: early acute right heart failure, early post-implant right heart failure, and late right heart failure (Hall et al. 2022). The variation in definitions, timing, type of device implanted, and RVF severity mean the incidence following LVAD implantation can range from 20.2 to 60.7% (Kapelios et al. 2022; Fitzpatrick et al. 2008; Matthews et al. 2008; Kormos et al. 2020; LaRue et al. 2017) (Supplementary Table 2).

## Clinical implications

Whatever definition is used, it is clear that RVD and/or RVF is associated with a significant range of short- and long-term complications across these high-risk cohorts. Following cardiac surgery, RVF is associated with increased post-operative (up to 30 days) mortality, ranging from 22 to 90% dependent on diagnostic criteria and population (Schuuring et al. 2013; Denault et al. 2016; Reichert et al. 1992; Moazami et al. 2004; Maslow et al. 2002). This increased risk is observed to persist to 2 years postoperatively where patients with RVF (*RVEF* < 20%) had a 16.7% mortality in comparison to 4.1% in those with normal RVEF. Consistent with the increased mortality, there is also a significant burden of post-operative morbidity, with increased duration of mechanical ventilation, renal dysfunction, and prolonged ICU and hospital stay (Levy et al. 2021; Maslow et al. 2002; Bootsma et al. 2018).

In those who have undergone cardiac transplantation, isolated RVF (PGD-RV) is associated with similar 18-month survival (approximately 55%^1^) to isolated PGD-LV but occurs more than five times as frequently (9.9% vs 1.7%) (Cosío Carmena et al. 2013). It is also associated with short-term mortality and increased requirement for post-operative renal replacement therapy(RRT) (Kaveevorayan et al. 2023). Following LVAD implantation, RVF is associated with significant post-operative morbidity; with longer hospital length of stay, longer duration of mechanical ventilation, increased frequency of post-operative bleeding, renal dysfunction, and increased requirement for RRT (Kormos et al. 2020; Matthews et al. 2008). Beyond the immediate post-operative period, RVF following LVAD implantation is associated with increased mortality at 1 and 2 years and with significant morbidity in the form of heart failure readmissions and gastrointestinal bleeding (Kapelios et al. 2022; Kormos et al. 2020; LaRue et al. 2017).

## Conclusion

We applaud the POQI-IX collaborators for robustly addressing the challenge of perioperative RV function. Our current understanding of this field is hampered by a paucity of clinical literature and conflicting definitions. What limited data we have however suggests a significant incidence and profound clinical impact such that these manuscripts should serve as a call to arms to examine this issue more comprehensively. Greater consensus regarding the definition of RVD and RVF is needed to advance the field both generally and in the perioperative period. Clinical decision tools such as the proposed POQI-IX ‘Individualized Right Heart Risk Assessment Tool (PIRRAT)’ (Ibekwe et al. 2023) have real promise to improve recognition of patients at risk of postoperative RVD but require appropriate clinical validation before their use can be advocated. Ultimately, however, we must progress to asking (and indeed answering) the most important question; if (as many in the field believe) postoperative RVD is a common and underappreciated contributor to postoperative morbidity and mortality, what can be done to mitigate this risk and improve patient outcome? As described here, potential, avoidable risk factors do exist, and as detailed in the POQI-IX ‘perioperative management of the vulnerable and failing right ventricle’ manuscript (Arora et al. 2023), potential supportive therapies are available. What we do not have but urgently need, therefore, are clinical trials of preventative strategies targeted at increased risk patients in appropriate surgical settings. There is much to understand, study, and trial in this area, but importantly for our patients, we are increasingly recognising the importance of these uncertainties.

### Supplementary Information

**Supplementary Material 1**

## Data Availability

No datasets were generated or analysed during the current study.

## Abbreviations

BCIS: Bone cement implantation syndrome
COPD: Chronic obstructive pulmonary disease
CPB: Cardiopulmonary bypass
DBD: Donation following brain death
DCD: Donation following circulatory death
IHD: Ischaemic heart disease
ISHLT: International Society for Heart and Lung Transplantation
LV: Left ventricle
LVAD: Left ventricular assist device
OLV: One-lung ventilation
OSA: Obstructive sleep apnoea
PAH: Pulmonary arterial hypertension
PGD: Primary graft dysfunction
POQI: Perioperative Quality Initiative
PAP: Pulmonary artery pressure
PMI: Perioperative myocardial injury
PE: Pulmonary embolism
PVR: Pulmonary vascular resistance
RRT: Renal replacement therapy
RV: Right ventricle
RVEF: Right ventricular ejection fraction
RVF: Right ventricular failure
RVAD: Right ventricular assist device
RVD: Right ventricular dysfunction

## Declarations

### Declarations

**Ethics approval and consent to participate**

Not applicable. This is a narrative review article reporting on previously published work.
