# Supplementary material for: Epidemiology of perioperative RV dysfunction: risk factors, incidence, and clinical implications
Source: Perioper Med (Lond). 2024 Apr 25;13:31. doi: 10.1186/s13741-024-00388-6 (PMC11046908; doi:10.1186/s13741-024-00388-6)
Supplement: Supplementary file 1 — Supplementary Material 1 [file 13741_2024_388_MOESM1_ESM.docx]

**Supplementary Table 1S: Additional references describing the incidence and clinical sequalae of perioperative right ventricular dysfunction in patients undergoing thoracic surgery.**

| **Study** | **Surgical population** | **N** | **Age (SD)**  **Proportion male (%)** | **Method of assessment** | **Definition of RVD** | **Postoperative change** | **Clinical significance / comments** |
| --- | --- | --- | --- | --- | --- | --- | --- |
| Reed et al, 1993 (1) | Thoracic  (predominantly lobectomy) | 10 | 64  50% | PAC | RVEF  RVEDV | Preop vs 24h postop:  RVEF: 0.42 (0.01) to 0.39 (0.02) (p=NS)  RVEDV increased (p<0.05) | Not examined |
| Reed et al, 1996 (2) | Thoracic  (lobectomy and pneumonectomy) | 35 | Not stated | PAC | RVEF  RVEDV | Pre-op vs POD1 vs POD2:  RVEF: 0.45 (0.01) to 0.39 (0.01) to 0.37 (0.01)  RVEDV: 90 (2) to 107 (3) to 103 (4) | Not examined |
| Backlund et al, 1998 (3) | Thoracic  (all thoracotomy) | 24 | 64 (9) ^A^  59 (10) ^A^  Sex not stated | PAC | RVEF  RVEDV | Pre-op vs POD2:  RVEF: 44 (4) to 37 (8) (NS) and ^A^ 38 (3) to 32(5) (p<0.05)  RVEDVI: 109 (13) to 116 (27) (NS) and ^A^ 126 (21) and 124 (26) (NS) | Not examined |
| Mageed et al, 2005 (4) | Thoracic  (lobectomy) | 30 | Not stated | PAC | RVEF  RVEDV | Pre-op vs 2h post-op:  RVEF: 37±6.63 to 27 (12.74) (p<0.05)  RVEDVI: 160 (38.51) to 141 (49.1) (NS) | Not examined |
| Elrakhawy et al, 2018 (5) | Thoracic  (lobectomy and pneumonectomy) | 178 | 66.3 (7.4)^A^  62.9 (9.7)^A^  69% | PAC | RVEF  RVEDV | Preop vs 48h post-op (pneumonectomy):  RVEF: 46.7 (6.3) to 42.3 (7.6), RVEDVI: 82.7 (7.6) to 109.4 (6.9)  Preop vs 48h post-op (lobectomy):  RVEF: 49.6 (9.8) to 42.3 (7.6), RVEDVI: 79.8 (8.4) to (95.5 (8.2)  P<0.001 for all | Not examined |
| Steffen et al(6) 2018 | Thoracic (predominantly lung resection) | 50 | 61.2 (12.4)  58% | TTE | RVGLS  RVFWLS  FAC  TAPSE | No change on POD7 | Not examined |

^A^Data presented for two experimental groups separately.

FAC – fractional area change; NS – non-significant; PAC - pulmonary artery catheter; PAP – pulmonary artery pressure; POD – postoperative day; RVD – right ventricular dysfunction; RVEDV(I) – RV end-diastolic volume (index); RVEF – RV ejection fraction; TAPSE – tricuspid annular plane systolic excursion; TOE - transoesophageal echocardiography; transthoracic echocardiography.

**Supplementary table 2: Summary of selected studies describing the incidence and clinical implications of right ventricular dysfunction following cardiac transplantation and LVAD implantation.**

| **Study** | **Surgical population** | **N** | **Age**  **Proportion male (%)** | **Method of assessment** | **Definition of RVD** | **Incidence of RVD** | **Clinical significance / comments** |
| --- | --- | --- | --- | --- | --- | --- | --- |
| Cosío Carmena et al, 2013 (7) | Cardiac Transplant | 857 | 53.3 (11.5)  53.8 (11.7)  643 (75.0%) | Clinical | Severe impairment of RV function, or both ventricles as assessed by direct visualization or by echocardiography, with haemodynamic compromise (SBP<90mmHg and/or CI<2.2L/min/m2), requiring >2 vasoactive drugs, or MCS with IABP or VAD within first 24 hours with absence of any other obvious cause. | 85 (9.9%) had isolated RV failure and 88 (10.2%) as part of biventricular failure. | In post-op PGD, the right ventricle was almost always affected (92%).  Biventricular had worst 18 month survival. Isolated PGD-RV Associated with similar 18 month survival as isolated PGD-LV but much higher incidence. |
| Nicoara et al, 2017 (8) | Cardiac Transplant | 317 | 36 [26- 46.0]  74.1% | ISHLT criteria^A^ | Requirement for mechanical support with right VAD implantation | 39 patients (12.3%) had evidence of PGD-RV  -17 (5.4%) isolated PGD-RV  -22 (6.9%) part of biventricular PGD | Most stringent ISHLT criterion^A^ for PGD-RV; need for RVAD.  May be more patients with clinically significant RV dysfunction  No clinical associations looking at PGD-RV |
| Singh et al, 2019 (9) | Cardiac Transplant | 450 | 47 [33-61]^B^  46 [32-60]^B^  348 (77.3%) | ISHLT criteria^A^ | PGD was defined using the 2014 ISHLT Consensus | Six patients (1%) developed PGD-RV | No clinical associations explored with PGD-RV |
| Kaveevorayan et al, 2023 (10) | Cardiac Transplant | 111 | 41 [25-57]  71.2% | PAC / clinical | Modified ISHLT criteria - elimination of the criteria for cardiac index in the presence of inotropic support, and expanding time to 72h after OHTx (Primary Isolated RV Failure [PI-RVF])^C^ | 65 patients (59%) had evidence of PI-RVF | Associated with post-operative mortality and requirement for RRT |
| Fitzpatrick et al, 2008 (11) | LVAD implantation | 266 | 52.0 (13.9)^B^  51.5 (11.6)^B^  210 (78.9%) | Clinical | RVAD implantation | 99 (37.2%) | No clinical outcomes reported. |
| Matthews et al, 2008 (12, 13) | LVAD implantation | 197 | 156 (79.2%) | Clinical | 1) intravenous inotrope support for >14 days  2) iNO for ≥ 48 hours  3) Right-sided circulatory support (ECMO or  RVAD)  4) hospital discharge with an IV inotrope. | 68 (34.5%) | More requirement for ventilatory support and RRT |
| Kormos et al, 2010 (14) | LVAD implantation | 484 | 376 (77.7%)  51.8 (13.5)^B^  51.0 (13.3)^B^  55.0 (11.0)^B^  48.6 (12.0)^B^ | Clinical | 1) Need for RVAD  2) Continuous inotropic support for at least 14 days  3) Late inotropic support starting after 14 days  1&2 combined – to form “early RVF.” | Total 98 (20.2%)  Subgroups  1 – 30 (6.1%)  2 – 35 (7.2%)  3 – 33 (6.8%) | RVF associated with longer hospital stay, bleeding, CPB time, renal dysfunction and lower 1 year survival (78% [no RVF] vs 59% [early RVF]) |
| LaRue et al, 2017 (15) | LVAD implantation | 445 | 359 (81%) | Clinical | Duration of inotrope  Requirement   - Mild (≤ 7 days) - Moderate (8–14 days) - Severe (>14 days)   *and*   - Severe acute (RVAD implantation) | 270 (60.7%) required inotropes for >7days  139 (31.2%) had “severe” RVF (Combined severe and severe acute). | 2-year mortality   - Mild 22.3% - Moderate 26.7% - Severe 47% - Severe (RVAD) 68.6%   Time to (composite); death, HF admission or GI bleed lower in severe group |
| Kapelios et al, 2020 (16) | LVAD implantation | 5537 | 4340 (78.6%) | Haemodynamic and clinical | Symptoms or findings of  persistent RV failure characterized by both:  Documentation of elevated central venous pressure (CVP) AND (clinical) Manifestations of elevated CVP | 1329 (30.6%) at one month, of these, RHF persisted in 5.3% at 12 months | Patients with persistent RHF at 3 months had the lowest 2-year survival (57%) while patients with de novo RHF or RHF which resolved by 3 months had more favourable survival outcomes (75% and 78% at 2 years, respectively; P<0.001) |

Presented as n (%), mean (standard deviation), median [inter-quartile range] or median (17).

RVF – RV failure; CI – Cardiac Index; MCS – mechanical circulatory support; IABP – intra aortic balloon pump; (R or L) VAD – (right or left) Ventricular Assist Device; PGD-(RV or LV) – primary graft dysfunction-(right ventricle or left ventricle); ISHLT - International Society of Heart and Lung Transplant; PAC – pulmonary artery catheter; OHTx – Orthotopic Heart Transplant; iNO – inhaled nitric oxide; ECMO – extra corporeal membrane oxygenation; RRT – renal replacement therapy; LVAD – left ventricular assist device; IV – intravenous; CPB – cardiopulmonary bypass; HF – heart failure; GI – gastrointestinal; CVP – central venous pressure

^A^PGD-right ventricle (PGD-RV): Within 24 hours of transplant. Diagnosis requires either both i and ii, or iii alone: i. Hemodynamics with RAP > 15 mmHg, PCWP < 15 mm Hg, CI<2.0 L/min/m2 ii. TPG < 15 mmHg and/or pulmonary artery systolic pressure < 50 mmHg, or iii. Need for RVAD

^B^Data presented for separate experimental groups.

^C^Mild PI-RVF: All the following (3 of 3): (1) RAP>15 mmHg, (2) PCWP<18 mmHg, (3) CI<2.0 L/min/m2 or off inotrope or requiring low dose inotropes for <72h post-transplant and (1) TPG<15 mmHg and/or (2) PASP<50 mmHg. Moderate PI-RVF: All the following (3 of 3): (1) RAP>15 mmHg, (2) PCWP<18 mmHg, (3) CI< I<2.0 L/min/m2 or escalating of inotrope requirements or inability to wean inotropes>72 h post-transplant and (1) TPG<15 mmHg and/or (2) PASP<50 mmHg. Severe PI-RVF: The need for RVAD or ECMO

**References**

**References**

1. Reed CE, Dorman BH, Spinale FG. Assessment of right ventricular contractile performance after pulmonary resection. Ann Thorac Surg. 1993;56(3):426-31.

2. Reed CE, Dorman H, Spinale FG. Mechanisms of Right Ventricular Dysfunction After Pulmonary Resection. Ann Thorac Surg. 1996;62:225-32.

3. Bäcklund M, Laasonen L, Lepäntalo M, Metsärinne K, Tikkanen I, Lindgren L. Effect of oxygen on pulmonary hemodynamics and incidence of atrial fibrillation after noncardiac thoracotomy. J Cardiothor Vasc An. 1998;12(4):422-8.

4. Mageed NA, El-Ghonaimy YAF, Elgamal M-AF, Hamza U. Acute effects of lobectomy on right ventricular ejection fraction and mixed venous oxygen saturation. Annals of Saudi medicine. 2005;25(6):481-5.

5. Elrakhawy HM, Alassal MA, Shaalan AM, Awad AA, Sayed S, Saffan MM. Impact of Major Pulmonary Resections on Right Ventricular Function: Early Postoperative Changes. Heart Surg Forum. 2018;21(1):E009-e17.

6. Steffen HJ, Kalverkamp S, Zayat R, Autschbach R, Spillner JW, Hagendorff A, Hatam N. Is Systolic Right Ventricular Function Reduced after Thoracic Non-Cardiac Surgery? A Propensity Matched Echocardiographic Analysis. Ann Thorac Cardiovasc Surg. 2018;24(5):238-46.

7. Cosío Carmena MD, Gómez Bueno M, Almenar L, Delgado JF, Arizón JM, González Vilchez F, Crespo-Leiro MG, Mirabet S, Roig E, Pérez Villa F, Fernández-Yañez JF, Lambert JL, Manito N, Fuente L, Sanz Julve ML, Pascual D, Rábago G, Millán I, Alonso-Pulpón LA, Segovia J. Primary graft failure after heart transplantation: characteristics in a contemporary cohort and performance of the RADIAL risk score. J Heart Lung Transplant. 2013;32(12):1187-95.

8. Nicoara A, Ruffin D, Cooter M, Patel CB, Thompson A, Schroder JN, Daneshmand MA, Hernandez AF, Rogers JG, Podgoreanu MV, Swaminathan M, Kretzer A, Stafford-Smith M, Milano CA, Bartz RR. Primary graft dysfunction after heart transplantation: Incidence, trends, and associated risk factors. Am J Transplant. 2018;18(6):1461-70.

9. Avtaar Singh SS, Banner NR, Rushton S, Simon AR, Berry C, Al-Attar N. ISHLT Primary Graft Dysfunction Incidence, Risk Factors, and Outcome: A UK National Study. Transplantation. 2019;103(2):336-43.

10. Kaveevorayan P, Tokavanich N, Kittipibul V, Lertsuttimetta T, Singhatanadgige S, Ongcharit P, Sinphurmsukskul S, Ariyachaipanich A, Siwamogsatham S, Thammanatsakul K, Sritangsirikul S, Puwanant S. Primary isolated right ventricular failure after heart transplantation: prevalence, right ventricular characteristics, and outcomes. Scientific Reports. 2023;13(1):394.

11. Fitzpatrick JR, Frederick JR, Hsu VM, Kozin ED, O'Hara ML, Howell E, Dougherty D, McCormick RC, Laporte CA, Cohen JE, Southerland KW, Howard JL, Jessup ML, Morris RJ, Acker MA, Woo YJ. Risk Score Derived from Pre-operative Data Analysis Predicts the Need for Biventricular Mechanical Circulatory Support. The Journal of Heart and Lung Transplantation. 2008;27(12):1286-92.

12. Matthews JC, Koelling TM, Pagani FD, Aaronson KD. The Right Ventricular Failure Risk Score: A Pre-Operative Tool for Assessing the Risk of Right Ventricular Failure in Left Ventricular Assist Device Candidates. Journal of the American College of Cardiology. 2008;51(22):2163-72.

13. Maslow AD, Regan MM, Panzica P, Heindel S, Mashikian J, Comunale ME. Precardiopulmonary bypass right ventricular function is associated with poor outcome after coronary artery bypass grafting in patients with severe left ventricular systolic dysfunction. Anesth Analg. 2002;95(6):1507-18, table of contents.

14. Kormos RL, Antonides CFJ, Goldstein DJ, Cowger JA, Starling RC, Kirklin JK, Rame JE, Rosenthal D, Mooney ML, Caliskan K, Messe SR, Teuteberg JJ, Mohacsi P, Slaughter MS, Potapov EV, Rao V, Schima H, Stehlik J, Joseph S, Koenig SC, Pagani FD. Updated definitions of adverse events for trials and registries of mechanical circulatory support: A consensus statement of the mechanical circulatory support academic research consortium. J Heart Lung Transplant. 2020;39(8):735-50.

15. LaRue SJ, Raymer DS, Pierce BR, Nassif ME, Sparrow CT, Vader JM. Clinical outcomes associated with INTERMACS-defined right heart failure after left ventricular assist device implantation. J Heart Lung Transplant. 2017;36(4):475-7.

16. Kapelios CJ, Lund LH, Wever-Pinzon O, Selzman CH, Myers SL, Cantor RS, Stehlik J, Chamogeorgakis T, McKellar SH, Koliopoulou A, Alharethi R, Kfoury AG, Bonios M, Adamopoulos S, Gilbert EM, Fang JC, Kirklin JK, Drakos SG. Right Heart Failure Following Left Ventricular Device Implantation: Natural History, Risk Factors, and Outcomes: An Analysis of the STS INTERMACS Database. Circulation: Heart Failure. 2022;15(6):e008706.

17. Ariti CA, Cleland JGF, Pocock SJ, Pfeffer MA, Swedberg K, Granger CB, McMurray JJV, Michelson EL, Östergren J, Yusuf S. Days alive and out of hospital and the patient journey in patients with heart failure: Insights from the Candesartan in Heart failure: Assessment of Reduction in Mortality and morbidity (CHARM) program. Am Heart J. 2011;162(5):900-6.
